# Analysis of the Role of *Bradysia impatiens* (Diptera: Sciaridae) as a Vector Transmitting Peanut Stunt Virus on the Model Plant *Nicotiana benthamiana*

**DOI:** 10.3390/cells10061546

**Published:** 2021-06-18

**Authors:** Marta Budziszewska, Patryk Frąckowiak, Aleksandra Obrępalska-Stęplowska

**Affiliations:** Department of Molecular Biology and Biotechnology, Institute of Plant Protection—National Research Institute, Władysława Węgorka 20, 60-318 Poznań, Poland; m.budziszewska@iorpib.poznan.pl (M.B.); p.frackowiak@iorpib.poznan.pl (P.F.)

**Keywords:** virus transmission, cucumovirus, fungus gnat, plant-insect-virus interactions, virus detection, virus vector

## Abstract

*Bradysia* species, commonly known as fungus gnats, are ubiquitous in greenhouses, nurseries of horticultural plants, and commercial mushroom houses, causing significant economic losses. Moreover, the insects from the *Bradysia* genus have a well-documented role in plant pathogenic fungi transmission. Here, a study on the potential of *Bradysia impatiens* to acquire and transmit the peanut stunt virus (PSV) from plant to plant was undertaken. Four-day-old larvae of *B. impatiens* were exposed to PSV-P strain by feeding on virus-infected leaves of *Nicotiana benthamiana* and then transferred to healthy plants in laboratory conditions. Using the reverse transcription-polymerase chain reaction (RT-PCR), real-time PCR (RT-qPCR), and digital droplet PCR (RT-ddPCR), the PSV RNAs in the larva, pupa, and imago of *B. impatiens* were detected and quantified. The presence of PSV genomic RNA strands as well as viral coat protein in *N. benthamiana*, on which the viruliferous larvae were feeding, was also confirmed at the molecular level, even though the characteristic symptoms of PSV infection were not observed. The results have shown that larvae of *B. impatiens* could acquire the virus and transmit it to healthy plants. Moreover, it has been proven that PSV might persist in the insect body transstadially. Although the molecular mechanisms of virion acquisition and retention during insect development need further studies, this is the first report on *B. impatiens* playing a potential role in plant virus transmission.

## 1. Introduction

*Bradysia* species, commonly referred to as mushrooms fly, nuisance fly, black fungus gnat, or dark-winged fungus gnat, belong to the Sciaridae family and are considered to be harmful in greenhouses [1]. These insects are especially ubiquitous in nurseries of horticultural plants, such as cucumber, tomatoes, ornamental plants productions, and edible mushrooms, causing significant damages and thus economic loss. Sciarid larvae are known to be polyphagous. Primarily, they feed on algae, fungi, decomposed plant debris, but they have also been reported to attack healthy plants (i.e., seedlings of carnations, cucumber, as well field plants like soybean, wheat, corn, pea) by feeding on roots and stems of young seedlings [2,3,4,5], or occasionally on leaf tissues and cuttings [5,6].

The life cycle of *Bradysia* spp. lasts 15–49 days, depending on the environmental conditions, and includes eggs, four larval instars, the pupal stage, and imagoes [7]. Adult females deposit eggs usually very close to the stem of young plants [3,8], which may be of importance for possible pathogen transmission. Herbivorous larvae cause physical damages to the stem and root tissues, disturbing water and nutrients uptake, which, in consequence, leads to wilting, stunting [3], chlorosis, premature foliage loss, or eventually, death [9,10]. Larvae may also affect plants indirectly, by allowing the entry of soil-borne pathogens. Notably, fungus gnats are known to be vectors of fungal pathogens. The role of *Bradysia* genus representatives in the transmission of oomycete and fungi, such as *Pythium* spp., *Fusarium* spp., and *Verticillium* spp., is well documented [11,12,13,14,15]. In 2017, Park et al. characterized the microbes of *B. agrestis*, indicating that this insect was associated with fungal and bacterial species. The dominant genera were *Bacillus*, *Rhodococcus*, and *Pseudomonas*, but only the latter might be dangerous for the agricultural environments in favourable conditions [16].

The viral gene expression data, obtained in our previous experiments on *N. benthamiana*-virus interactions, indicated the PSV genes were detectable in some healthy plants (non-infected), suggesting accidental transmission of the virus from infected plants to remote healthy plants growing inside the same cabin. The only factor that might have contributed to these infections were insects of *B. impatiens*, incidentally brought to strictly controlled glasshouse cabins, most probably with the soil. In the absence of other potential vectors, we hypothesized that these insects might be the agents transmitting the viral pathogen to healthy plants.

Peanut stunt virus (PSV) belongs to the *Cucumovirus* genus in the family *Bromoviridae*. It is a severe pathogen distributed worldwide with a broad host range, mainly of legumes such as pea (*Pisum sativum* L.), bean (*Phaseolus vulgaris* L.), and yellow lupine (*Lupinus luteus* L.) [17]. Its genome (~8.3 kb in size) consists of three genomic positive-sense single-stranded RNAs and two subgenomic RNAs. RNA1 and 2 encode proteins 1a and 2a, respectively, which are components of the viral replication complex. RNA 3 encodes protein 3a, which is involved in the virus movement. Subgenomic RNA4 acts as a messenger RNA for the viral coat protein (CP), whereas RNA4A encodes protein 2b, known as the post-transcriptional gene silencing (PTGS) suppressor [18]. Some of the PSV strains, including the one used in our study, PSV-P, contain satellite RNA [19]. In the experimental conditions, PSV is mechanically transmissible via the plant sap. In contrast, in the field, it is known to be transmitted in a non-persistent manner by several aphid species and mechanically [14,17,20].

This study is aimed at verification of the hypothesis of the potential involvement of *B. impatiens* in the transmission of PSV. Using the two highly sensitive molecular biology tools, RT-qPCR, and RT-ddPCR, and a biological test based on the plant inoculation method, we have undertaken a study on the possibility of the virus acquisition by *B. impatiens*. Then, we explored PSV retention in the body of particular insect’s developmental stages and its further transmission to healthy plants.

The results have shown that *B. impatiens* might be considered as a vector of not only fungal phytopathogens, but also plant RNA viruses.

## 2. Materials and Methods

### 2.1. Virus-Free Insect Cultivation

For experimental purposes, the colony of *B. impatiens* originating from the slugs breeding was kindly provided by the Department of Entomology and Agricultural Pests, Institute of Plant Protection—NRI (Poznań, Poland). The larvae, pupae, and adults were cultivated in moist soil, in a 1-L plastic box with a perforated lid for ventilation. Larvae were fed on Chinese cabbage leaves or carrot roots. In the next step, 20 adult fungus gnats were collected by exhauster aspiration and placed on a Petri dish with 2% (*w*/*v*) agar medium, at room temperature, in the laboratory conditions. The females laid eggs, of which 100–150 were taken and applied directly to a fresh 2% (*w*/*v*) agar plate with a small brush. To confirm that insect material is PSV-free, the 3-day-old eggs (10 eggs/sample) were taken with a brush and placed in an Eppendorf tube, washed with sterile water, and stored in 70% ethanol at −80 °C for further RT-PCR detection with virus-specific primers (Table 1). Once the remaining eggs had hatched, the 2–3 second-instar larvae (about 4-day-old), and subsequently 2–3 3-day-old pupae and 2–3 adult insects were also collected with small brush and exhauster (for adults) and placed into Eppendorf tubes, as mentioned above, for RT-PCR tests.

### 2.2. The Insect Species Identification Using PCR

The taxonomic identification of cultivated fungus gnats belonging to the *Bradysia* genus was based on the sequence analysis of the I subunit of mitochondrial cytochrome c oxidase gene (*mtCOI*). For this purpose, the DNA was isolated from six samples, each containing a single adult insect, using a NucleoSpin Tissue Kit (Macharey-Nagel, Dueren Germany) followed by PCR reaction with primers, designed by Folmer et al. [20], and hybridizing to the fragment of *mtCOI* gene. PCR profiles were as follows: denaturation at 95 °C for 3 min, 35 cycles: 95 °C for 30 s, 45 °C for 30 s, 72 °C for 40 s, and final elongation at 72 °C for 5 min. Products of amplification were sequenced and submitted to the GenBank database with the following accession numbers MW798234, MW798235, MW798236, MW798237, MW798238, MW798239. The obtained results were analysed using the Nucleotide BLAST tool (https://blast.ncbi.nlm.nih.gov/Blast.cgi. The National Center for Biotechnology Information, NCBI, Bethesda, MD, USA).

### 2.3. Viral Strain and Plant Material

As a viral source, the peanut stunt virus strain P (PSV-P, from *Lupinus luteus* L.) from our laboratory collection was used [19]. PSV was maintained and propagated in *Pisum sativum.* To prepare inoculum for mechanical transmission, the infected pea plants, showing systemic symptoms, were homogenized in 0.05 M phosphate buffer, pH 7.5, and the sap was rubbed in the previously carborundum-dusted leaves of 3–4-week-old *N. benthamiana* seedlings [17]. The plants were grown in the greenhouse with a 16 h light/8 h dark cycle at 21/18 °C day/night temperature. Plants were observed for symptom development for 2–4 weeks after mechanical inoculation. After 10–14 days, the presence of PSV RNAs was verified in the systemic leaves by RT-PCR with specific primers (Table 1). Infected plants were used as a virus source for the transmission studies by fungus gnats. All biological experiments were conducted in the glasshouse of the Department of Molecular Biology and Biotechnology of the Institute of Plant Protection—National Research Institute (Poznań, Poland).

### 2.4. Studies on the Fungus Gnats Ability to Acquire a Virus

**Figure 1:**
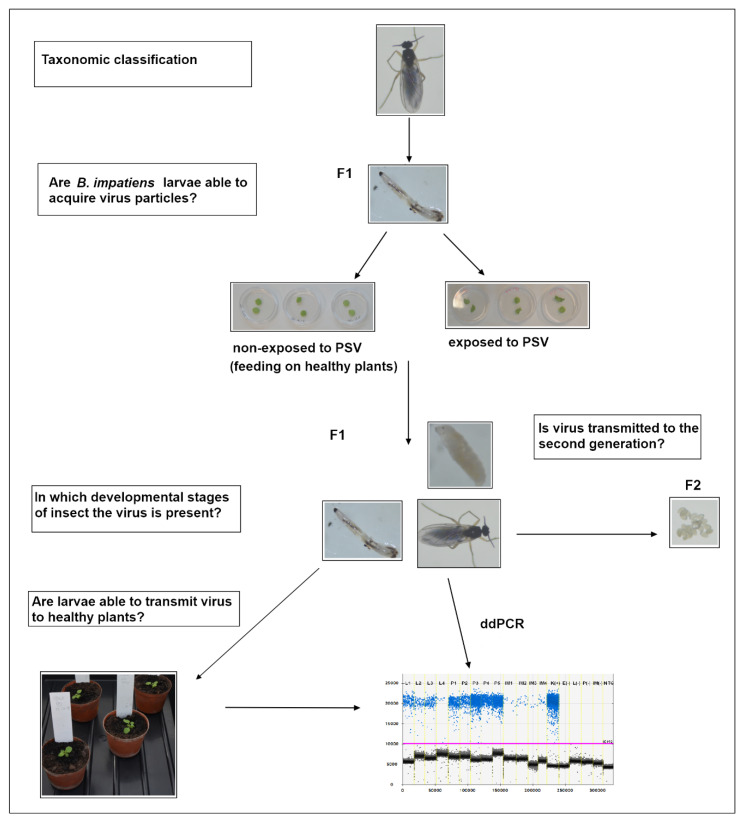
The scheme of experimental setup designed to analyse the *B. impatiens* larvae’s ability to acquire a virus, virus transstadial persistence, and the potential to spread the virus to healthy plants.

To analyse the potential of the fungus gnat to acquire the virus, we collected 30–40 larvae from a virus-free colony of *B. impatiens* (4-day-old) and placed them for 24 h, on a sterile Petri dish with 2% (*w*/*v*) agar medium and leaf disks of PSV-infected *N. benthamiana* plants, as a food source. In negative controls, 30–40 specimens of virus-free larvae were placed for 24 h on a separate Petri dish, with agar and disks of a healthy *N. benthamiana* (Figure 1). After this time, virus-exposed and non-exposed larvae were transferred separately to sterile plates with 2% (*w*/*v*) agar, according to Gardiner et al. [21], for the next 24 h. Then, 2–3 larvae of *B. impatiens*, PSV-exposed and non-exposed, were caught with a brush and put respectively in Eppendorf tubes with 70% ethanol and frozen at −80 °C, for further RT-PCR reactions with PSV-specific primers (Table 1). To verify the possibility of virus persistence at the insect’s developmental stages, 3-day-old pupae (2–3 specimens), and emerging adults (2–3 individuals), developed from virus-exposed and respectively virus-free larvae were collected. RT-PCR detection of PSV was subsequently performed. Additionally, the PSV detection and quantification analyses of viral RNAs in the single larva, pupa, and imago (from PSV-exposed and non-exposed specimens) were also conducted. For this purpose, 4–5 specimens of 4-day-old larvae, 3-day-old pupae, and newly emerged imago were caught and put individually in Eppendorf tubes for quantitative analyses using the digital droplet PCR (ddPCR) approach preceded by RT-qPCR-based virus detection. A virus acquisition study was performed in triplicate.

### 2.5. Virus Transmission Assay

After the acquisition access period (AAP), the virus-exposed larvae were transferred to a new, sterile Petri dish for 24 h [21], as was described above. Then, 5–7 randomly collected larvae were placed in a soil pot (a soil used in all biological tests was previously heat sterilised at 100–120 °C) near to the root of two-leaf stage *N. benthamiana* healthy seedlings, previously molecularly tested to exclude PSV infection. As negative controls, we used larvae feeding on healthy leaf disks and proceeded as described above. The analysis included five biological replicates.

The plants were kept in insect-rearing cages (BugDorm, Taichung, Taiwan) under controlled conditions, as was mentioned above. Three weeks post-inoculation, two leaf disks were collected from all tested plants, and the total RNA was isolated. Afterward, the virus RT-PCR detection followed by real-time PCR and ddPCR were performed (Figure 1).

### 2.6. Total RNA Isolation

Total RNA was isolated from virus-exposed or non-exposed insect samples and small two leaf disks (about 25 mg) of all tested *N. benthamiana* plants. Plant material, as well as whole insect body samples, were homogenized with a small plastic pestle. RNA isolation was carried out using a Tri-Reagent solution (Ambion, Life Technologies, Naugatuck, CT, USA), followed by 2-propanol precipitation. Total RNA from insect body and plant material was dissolved, respectively, in 10 μL and 20 μL of RNase-free water. The RNA quantity was assessed with spectrophotometer NanoDrop ND-1000 (Thermo Scientific, Lenexa, KS, USA) and analysed under non-denaturing conditions by electrophoresis. Aliquots were stored at −80 °C until use.

### 2.7. RT-PCR

For the reverse transcription step, 0.5 µg of total RNA from individual insect, 1 µg of total RNA from remaining insect samples, and 1 µg from plant tissue were combined with 200 ng of random hexamer primers and incubated at 65 °C for 5 min. Afterward, the mixture was cooled on ice and 4 µL of 5X Reaction Buffer, 2 µL of 10 mM dNTPs, 20 U/μL RiboLock RNase Inhibitor and 200 U/μL of RevertAid M-MuLV Reverse Transcriptase (Thermo Scientific, Lenexa, KS, USA) were added. The reaction was carried out in a total volume of 20 μL. The thermal profile was as follows: 10 min at 25 °C, 60 min at 42 °C, termination at 70 °C for 5 min. In the case of insects, the synthesized cDNAs were additionally amplified using PCR with *mtCOI* specific primers using above mentioned thermal conditions. The obtained cDNA served also as a template for PCR reactions with primers amplifying three PSV genes: 1a, 2b as well the fragment spanning the movement and coat protein (MPCP) (Table 1). PCR was performed in 15 μL of the reaction mixture that contained 1X DreamTaq Master mix (ThermoScientific, Lenexa, KS, USA), 0.5 µM primers, 1 µL of cDNA template (reverse transcribed on the total RNA isolated from 2–3 individuals) and sterile water to the final volume. Thermal profiles consisted of initial denaturation at 95 °C for 3 min followed by 40 cycles of denaturation at 95 °C for 30 s, annealing step at a temperature appropriate for used primer pairs (Table 1) for 30 s, elongation at 72 °C for 40 s, and final extension at 72 °C for 5 min. As positive controls, we used 1 µL of cDNA synthesized from 1 µg of total RNA isolated from the virus-infected *N. benthamiana* plant. The RT-PCR was electrophoretically analysed, and products of randomly chosen plant and insect samples were extracted from the gel using The Wizard^®^ SV Gel and PCR Clean-Up System (Promega, Madison, WI, USA). RT-PCR products were Sanger sequenced (Genomed S.A., Warsaw, Poland) and then analysed in the Nucleotide BLAST tool.

### 2.8. Virus Detection in Insect Individuals Using Real-Time PCR

The reaction was carried out in the 10 µL of reaction mixture containing 1X iTaq Universal SYBR Green Supermix (Bio-Rad, Hercules, CA, USA), 0.3 µM each reverse and forward primers q1F/q1R, q2aF/q2aR, qCPF/qCPR complementary, respectively, to the PSV 1a, 2a and CP gene (Table 1), 1 µL of cDNA template (transcribed on total RNA isolated from tested individual larva, pupa, imago, healthy and PSV-infected *N. benthamiana*), and the sterile water. The amplification was performed in a LightCycler 96 real-time thermocycler (Roche Applied Science, Mannheim, Germany) under the following cycling conditions: 95 °C for 3 min, followed by 40 cycles of 95 °C for 15 s, 55 °C for 30 s (Table 1) and 72 °C for 30 s. All samples were analysed in three technical replicates.

### 2.9. Quantitative Analysis of Viral RNAs Using Digital Droplet PCR (RT-ddPCR)

The digital droplet PCR (ddPCR) technique has provided an alternative method for absolute quantitation of nucleic acids based on fractionating each sample into water-oil emulsion before the PCR reaction that occurs within each droplet. The copy number of viral targets can be assessed by direct counting PCR-positive droplets among all reactions according to Poisson’s distribution. The reactions were performed using BioRad QX200 System (Bio-Rad, Hercules, CA, USA). The reaction mixture contained 1X QX200 ddPCR EvaGreen Supermix (Bio-Rad, Hercules, CA, USA), 0.25 µM of PSV specific primer mix (q1F/q1R, q2aF/q2aR) (Table 1), 1 µL of previously synthesized cDNA, and sterile water to a final volume of 20 µL. The cDNA of positive control was diluted 1x 10^−4^–10^−6^ (PSV- inoculated *N. benthamiana*). Vortexed samples were loaded into DG8^TM^ Cartridge for QX200 Droplet Generation (Bio-Rad) sample wells, and 70 µL of QX200 Droplet Generation Oil for Eva Green (Bio-Rad) was added into oil wells. The reaction mixture was then emulsified with Droplet Generation Oil for EvaGreen (Bio-Rad, Hercules CA, USA). Afterward, the prepared emulsions were carefully transferred into a 96-well plate, heat-sealed using a PX1™PCR Plate Sealer (Bio-Rad, Hercules, CA, USA), and proceed to thermal cycling using C1000 Thermal Cycler (Bio-Rad). The thermal conditions were as follows: an initial denaturation cycle of 10 min at 95 °C, followed by 40 cycles of denaturation for 30 s at 94 °C, annealing for 60 s at 57 °C (ramping rate set to 2 °C/s), and a final incubation for 10 min at 98 °C, ending at 4 °C. After amplification, the 96-well plate was placed into the Droplet Reader (Bio-Rad, Hercules, CA, USA). The reader measured the intensity of green fluoresce signals of each droplet and proceeded with the QuantaSoft™ analysis software version 1.7 (Bio-Rad). Positive droplets with higher fluorescent signals and negative droplets with lower fluorescent signals were divided by applying a fluorescence amplitude threshold. The absolute concentration of each sample was automatically reported by the ddPCR software by calculating the ratio of the positive droplets over the total droplets combined with Poisson distribution https://www.bio-rad.com/webroot/web/pdf/lsr/literature/Bulletin_6407.pdf. Thus, the final concentration of templates was equal to the results, as calculated by the software.

### 2.10. Western Blot Assay

Immunodetection of PSV coat protein in *N. benthamiana* plants was performed using Western blot analysis after the plant exposure to the virus-infected larvae of *B. impatiens*. Seven days after the contact with viruliferous larvae, one disc of the systemic leaf from each plant was taken. As a negative control, we used plant material from the healthy, untreated *N. benthamiana.* As a positive control, we used a leaf disc from *N. benthamiana* mechanically inoculated with PSV, as well as the two samples of purified particles of PSV-P from our collection. The tested plant samples were homogenized in 100 µL 2X Laemmli sample buffer. Two microliters of virus preparation were mixed with 8 µL of sterile water and 10 µL of 2X sample buffer. Then, all samples were denatured at 99 °C for 10 min, centrifuged at 16,500× *g* for 10 min to remove plant debris. The PSV CP protein was detected by Western blotting as follows: 15 µL of the protein lysates were separated in 12% sodium dodecyl sulphate-polyacrylamide gel electrophoresis (SDS–PAGE), followed by protein transfer onto a PVDF membrane. The filter was blocked for 1 h at room temperature with 5% non-fat milk in Tris-buffered saline with 0.1% Tween (TBS-T), followed by incubation (1 h at room temperature) with antiserum against native PSV virion preparations (produced in rabbit) (kindly provided by Prof. H. Pospieszny from The Department of Virology and Bacteriology of IPP-NRI, Poznań, Poland) at a dilution of 1:1000 in blocking buffer. The membrane was washed three times with TBS-T, followed by incubation (1 h at room temperature) with a secondary antibody conjugated with phosphate alkaline (goat anti-rabbit IgG, Sigma-Aldrich, Darmstadt, Germany) at a dilution of 1:25,000. Afterward, the membrane was washed three times with TBS-T and the reaction was developed with 1 mL of Western Blue^®^ Stabilized Substrate for Alkaline Phosphatase (Promega, Madison, WI, USA).

## 3. Results

### 3.1. Insect Taxonomic Classification

The species of a cultivated population of fungus gnats were analysed based on the DNA sequence of the *mtCOI* gene. The amplified and subsequently sequenced 681 bp fragments of the *mtCOI* gene of the fungus gnats were compared with *Bradysia* sequences already deposited in the GenBank database and showed 99–100% of nucleotide sequence identity to *Bradysia impatiens* species, also known as *B. difformis* or *B. paupera* [23].

### 3.2. PSV Acquisition by B. impatiens Larvae

**Figure 2:**
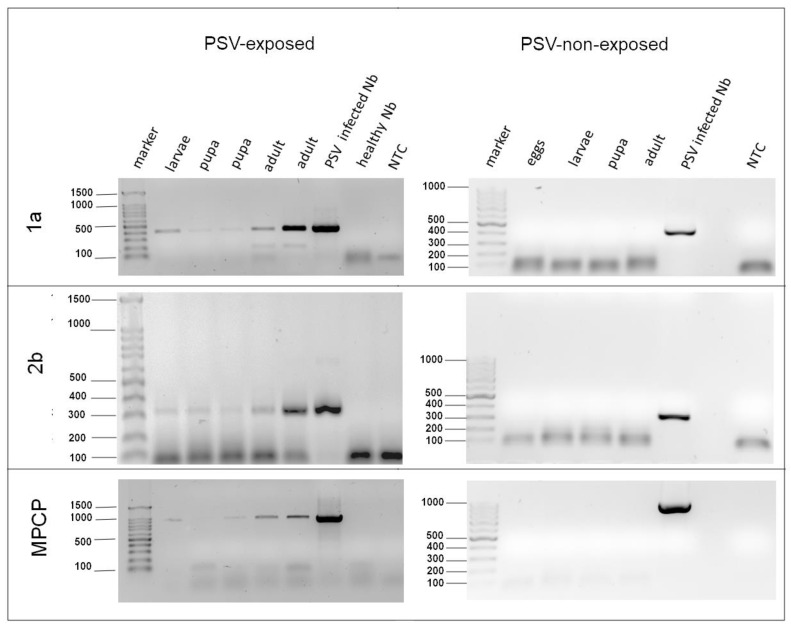
RT-PCR detection of peanut stunt virus RNAs in developmental stages of *Bradysia impatiens.* The RT-PCR products amplified with three PSV-specific primer pairs hybridizing to 1a, 2b, and fragment spanning 3a gene (movement protein, MP) and coat protein (CP); total RNA isolated from larvae, pupae, and adults (2–3 specimens) of *B. impatiens* population cultivated in laboratory conditions on Petri dishes were used as a template. M—marker Gene Ruler 100 bp (Thermo Scientific, Lenexa, KS, USA); NTC—no template control.

The exposure of *B. impatiens* larvae to PSV-infected plant tissue, followed by the RT-PCR test, confirmed the presence of PSV in fungus gnat’s body. Therefore, the possibility of PSV persistence in the subsequent insect developmental stages was also molecularly tested. The specific RT-PCR amplification products for viral genes 1a, 2b, and MPCP segment of respective size 426, 320, 958 bp were obtained in PSV-exposed larvae, and no products were observed in larvae not-exposed to the virus (Figure 2). The analysis of the virus persistence in the following developmental stages showed that both pupae and imago samples of *B. impatiens* maintained PSV as well, while in insects without previous contact with the virus (negative controls), the viral RNA was undetectable (Figure 2). Additionally, using the RT-PCR, we detected the specific products of the 1a PSV gene in the total RNA isolated from the second generation (F2) of eggs of *B. impatiens* (Figure S1). However, because there was no repeatability, this data remained inconclusive. Chosen amplicons of 1a, 2b, and MPCP region obtained for virus-exposed insects were Sanger sequenced. The nucleotide sequence analyses using BLAST revealed their high nucleotide sequence identity in the range of 98.5–99.2%, 99.66–100%, and 99.2–100%, to corresponding PSV-P RNAs, previously deposited in the GenBank (NCBI, Bethesda, MD, USA) under accession numbers EU570236 (RNA1), EU570237 (RNA2), and EU570238 (RNA3), respectively.

### 3.3. Analysis of Virus RNA Accumulation in Individuals of Fungus Gnats at Various Developmental Stages

**Figure 3:**
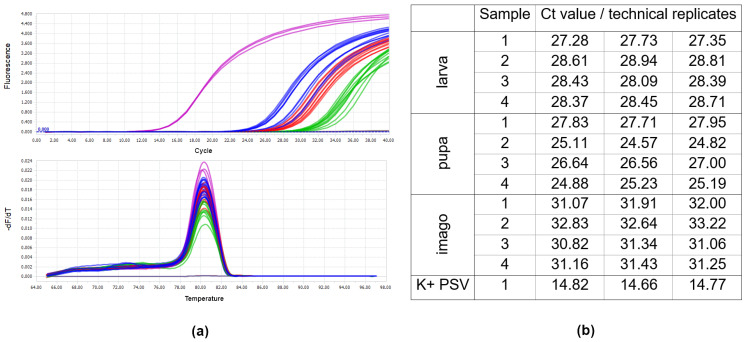
Results of PSV detection by RT-qPCR in single specimens of larva, pupa, and imago of *B. impatiens* after the virus acquisition by larvae feeding on PSV-infected *N. benthamiana* plants. Panel (**a**) The amplification plots of PSV 1a gene detected in a single larva—red line, pupa—blue line, imago—green line; positive control (PSV-infected *N. benthamiana*)—pink line (upper). Below, melting (dissociation) curves for amplicons of the PSV ORF 1a (replicase gene). For each developmental stage of fungus gnat, four single specimens were analysed, in three replicates. No amplification plots were obtained in the case of negative controls, including healthy larva, pupa, and imago, which were not exposed to PSV as well in NTC—no template control—grey line. Panel (**b**) Table with Ct values obtained by using in RT-qPCR analysis for PSV 1a gene for viruliferous larvae, pupae, and imago (four samples). Provided data concern the PSV-exposed larvae, pupae, and imago, which developed from PSV-exposed larvae, in three replicates. The Ct value for the positive control, the PSV-infected *N. benthamiana* (K + PSV), is also included.

**Figure 4:**
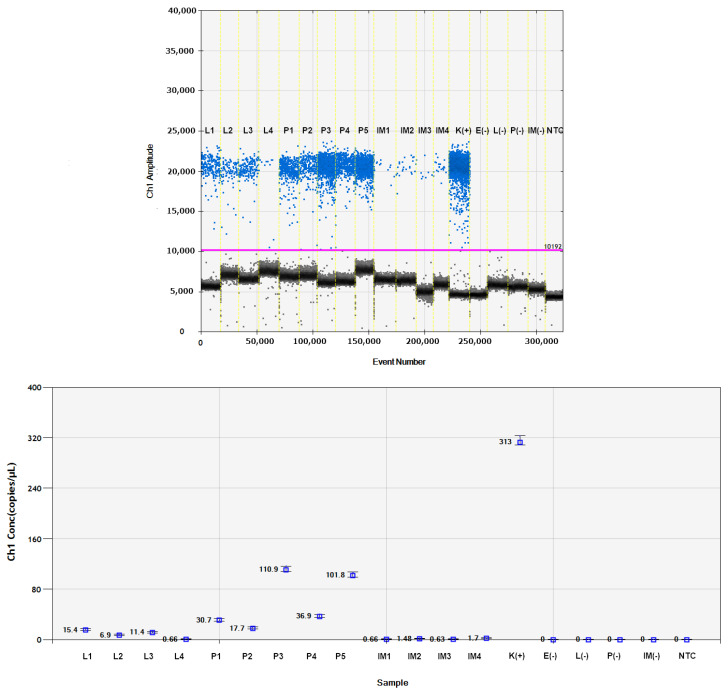
Detection of PSV 1a gene in the subsequent developmental stages of *B. impatiens* using EvaGreen digital droplet PCR approach. (**Upper**): 1-D plot diagram presents differences in amplitudes of fluorescence signals between positive and negative droplets. Blue dots: positive droplets with amplification, grey dots: negative droplets with no amplification. Pink line: threshold separating population of negative from positive droplets. (**Below**): the diagram showing the concentration of viral copies per 1 µL of the reaction mixture. L—larva, P—pupa, IM—adult stage, E—egg from a virus-free *B. impatiens* colony, (−)—negative control samples: PSV-non-exposed, NTC—no template control, K(+) —positive control, *N. benthamiana* plants infected with PSV inoculum.

The virus was detected in two successive developmental stages of *B. impatiens* obtained from larvae previously exposed to PSV-infected plants. No amplification products were obtained for the control *B. impatiens* specimens fed on healthy plants. The melting peaks of the amplified fragment of the PSV 1a gene in the PSV-infected *N. benthamiana* (positive control) coincided with the dissociation curves of analysed insect samples exposed to PSV (Figure 3). The obtained Ct values for pupae reached the highest values between 24 and 27, in the case of larva from 27 to 28, whereas the Ct for imago individuals were registered between 30–33 (Figure 3). Quantitative analyses based on ddPCR indicated that the highest copy number of viral gene per 1 µL of the reaction mixture was detected for pupae samples, a bit lower in the case of larvae, whereas in imago specimens, the level of virus RNA accumulation was the lowest in comparison to the remaining developmental stages (Figure 4).

### 3.4. Bradysia impatiens Ability to Vector PSV to Healthy Plants

To verify the virus vectoring ability of *B. impatiens,* the larvae which fed on PSV-inoculated leaves and parallel on the virus-free plant were put into soil pots with young *N. benthamiana*. It is worth adding that, before the transmission assay, all plant seedlings were molecularly tested to exclude PSV infection.

**Figure 5:**
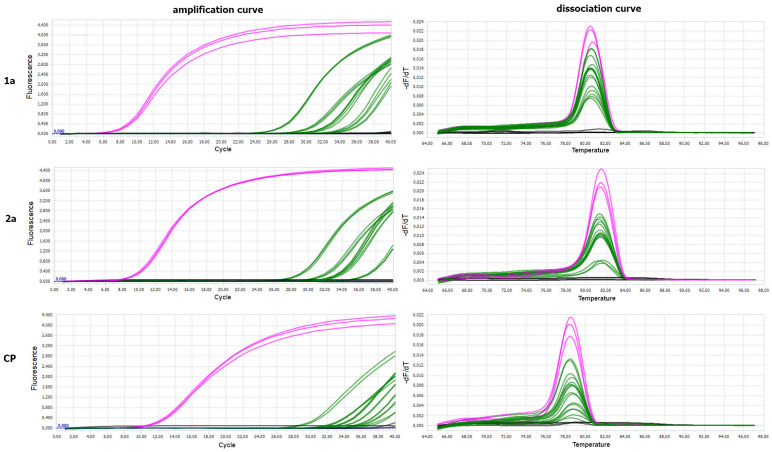
The RT-qPCR detection of PSV RNAs in *N. benthamiana* plants treated with PSV-exposed larvae of *B. impatiens*. The diagrams present the amplification (**left**) and dissociation curves (**right**) for amplification products of 1a, 2a, and CP PSV genes, detected in *N. benthamiana* plants; pink line—PSV-inoculated *N. benthamiana* (positive control); green lines—tested *N. benthamiana* plants treated with PSV-exposed larvae; black line—negative controls, healthy plant as well as NTC—no template control.

The plant’s visual inspection, three weeks after the inoculation by PSV-infected *B. impatiens* larvae, did not reveal any characteristic disease symptoms like chlorosis, mosaics, stunting, or leave malformations. Thus, they were tested for the presence of viral RNAs using molecular tools. PSV detection was performed utilizing RT-qPCR. The analyses were based on the amplification of three viral targets 1a, 2a, and CP genes. The melting peaks of the amplified fragments for PSV-infected *N. benthamiana* (positive control) coincided with the dissociation curves obtained for tested *N. benthamiana* plants, previously treated with PSV-exposed *B. impatiens* larvae. No amplification plots were observed for healthy control plants (Figure 5).

**Figure 6:**
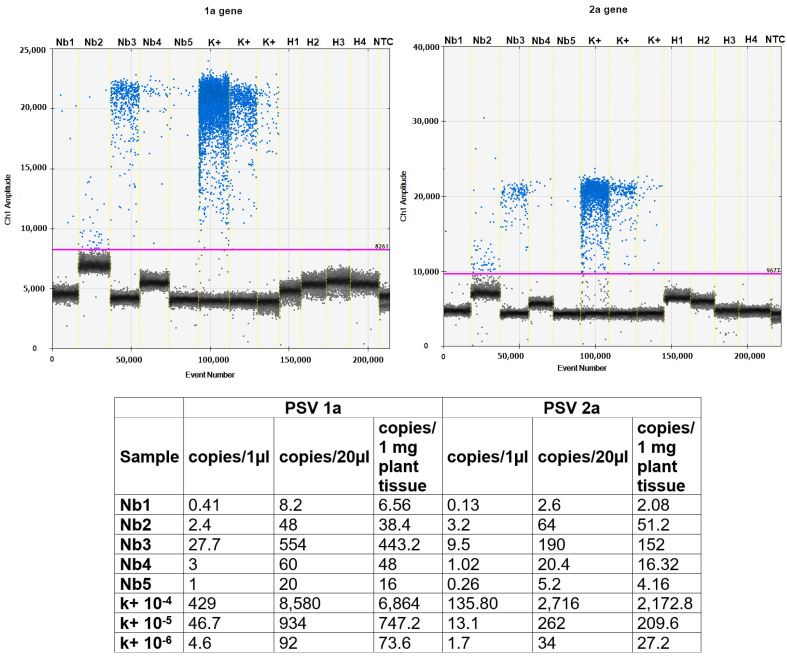
The ddPCR detection of viral 1a and 2a genes in *N. benthamiana* plants after inoculation with PSV-exposed larvae. (**Upper**) 1-D plot diagrams present differences in amplitudes of fluorescence signals between positive and negative droplets for 1a (**left**) and 2a (**right**). The black droplets represent negative droplets with no templates, and blue represent positive droplets containing amplified templates; pink line: threshold separating negative from positive droplets. Nb1–Nb5 samples of tested plants inoculated with the viruliferous insect; H1–H4 healthy plants of *N. benthamiana*; K(+) positive controls cDNA from PSV infected plants (10—fold diluted, 1 × 10^−4^, 10^−5^, 10^−6^); NTC—no template control. (**Below**) The EvaGreen ddPCR absolute quantification of copies number of PSV 1a and 2a genes per 1 µL of ddPCR reaction mixture, in 20 µL of the reaction mixture and the equivalent of copy number of viral RNA1 and RNA2 per 1 mg of plant tissue.

The absolute quantification, based on 1a and 2a viral targets, was performed using a ddPCR reaction. EvaGreen ddPCR results showed that the accumulation of the 1a gene is higher than the 2a gene. The EvaGreen ddPCR showed positive droplets for tested plants detecting 20–554 copies of 1a gene in the 20 µL reaction mixture that corresponds to 16–443 copies per 1 mg plant tissue. The absolute quantity of 2a gene was a bit lower, indicating 20–190 copies in 20 µL of PCR volume, which corresponds to 16–152 copies/1 mg plant tissue in three out of five tested plants, and in the two remaining plants (Nb1 and Nb5), we indicated only ~3–5 copies/1 µL of reaction, the equivalent of 2–4 copies per 1 mg of plant tissue (Figure 6).

### 3.5. Detection of PSV by Western Blotting

**Figure 7:**
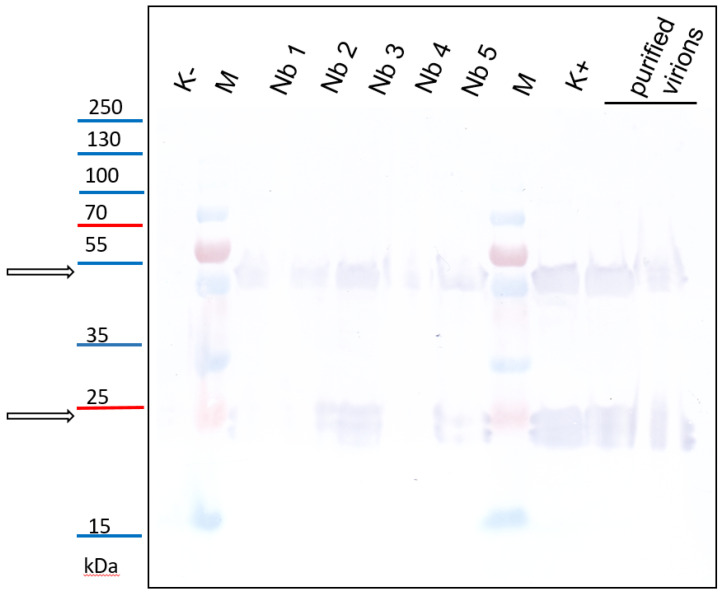
Detection of PSV coat protein in *N. benthamiana* plants inoculated with virus-exposed larvae of *B. impatiens* by western blotting. K−—healthy plant, untreated *N. benthamiana*; samples Nb1-5: *N. benthamiana* plants inoculated with *B. impatiens* larvae; M—protein marker PageRuler™ Prestained Protein Ladder Plus (Thermo Scientific, Lenexa, KS, USA), K+—positive control, PSV-inoculated plant; purified virions: as positive controls protein extracts from native virus preparations were also used. The PSV coat protein and its dimers are indicated by black arrows.

To finally confirm that PSV was transmitted by viruliferous larvae of *B. impatiens*, the PSV specific antisera was used for immunodetection. Western blot on crude plants extracts from *N. benthamiana* plants inoculated with PSV-infected larvae confirmed the presence of PSV coat protein. We detected the CP (ca. 24 kDa) and its dimers in plants inoculated with *B. impatiens* PSV-exposed larvae, the same signal was detected in the positive controls: *N. benthamiana* plants mechanically inoculated with PSV, and in purified virions preparations. No signal was detected for healthy, untreated *N. benthamiana* plants (Figure 7).

## 4. Discussion

The primary focus of this study was to determine the ability of *B. impatiens* larvae, in laboratory trials, to acquire and then to vector PSV. Our previous observations, performed in the controlled experimental conditions, indicated the accidental contamination with PSV of previously healthy plants that were grown in the same glasshouse cabin with virus-infected *N. benthamiana*. Thus, we have undertaken to analyse the potential involvement of *Bradysia* spp., which was the only factor present in greenhouse soil that could have contributed to the virus transmission. The RT-PCR analyses carried out on RNAs isolated from samples of larvae, pupae, and adults of fungus gnat, collected from soil pots, unexpectedly confirmed the presence of PSV genomic RNAs in all insect stages. These data prompted us to explore this observation.

Indeed, the analyses performed in this study, confirmed our preliminary results. We confirmed the ability of virus acquisition by the larval stage of *B. impatiens* and its persistence within the insect body during its development. The observation suggests that the virus persists transstadially. In this respect, these data are in agreement with the results of the experiments on fungi vectoring capacity of *B. impatiens* [15]. It is worth adding that, when we started work on this issue, we also detected the presence of PSV RNA in the second generation (F2) of *B. impatiens*, namely, in freshly laid eggs from adult females of *B. impatiens* (whose larval stadium of F1 was exposed to PSV) as well as in the specimens of larvae from the second generation (F2) hatched from these eggs (Supplementary Figure S1). However, we did not obtain repeatability, which may have resulted from the low level of virus RNA accumulation or possibly from the fact that PSV was not detectable in all laid eggs. That is why we cannot conclude that the virus persists transgenerationally in *B. impatiens*. However, these results showed that PSV-infested larvae developed into PSV-infested pupae and then into PSV-infested adults.

In the light of these data, a further question that arises is about the mode of virus transmission in the fungus gnat’s body. Thus far, cucumoviruses have been known to be transmitted by aphids (subfamily Aphidinae) in a non-persistent manner. Over 80 species of aphids have been identified to vector CMV [24,25], whereas PSV is known to be transmitted by *Aphis craccivora*, *A. solanella*, *A. spiraecola*, *Myzus persicae*, and *Liaphis erysimi* [26,27]. Currently, many authors focus on clarifying the cucumovirus-aphid-plant interactions [24,28,29,30]. Noteworthy, Kameya-Iwaki et al. have proven that PSV and CMV might be transmitted by *M. persicae* in a non-persistent but also in a semi-persistent manner, depending on the plant species used for the assay [25,31].

Viruses vectored by insects are divided into two categories: non-circulative (NC) and circulative viruses (CVs). In the first category, the viral particles attach to the cuticle of the insect vector without circulation in the insect body [32]. The NC viruses are divided into subcategories, transmitted in a non-persistent and semi-persistent manner, depending on the duration of virus retention in the insect’s body. Non-persistent virus transmission is characterized by very short acquisition and inoculation times of seconds to minutes. In the semi-persistent transmission, the acquisition can occur within minutes, but the transmission efficiency increases with prolonged insect feeding. Moreover, the second distinguishing feature is the retention period of hours to days [33]. In the second group, CVs need to circulate in the insect tissue before transmission to healthy plants. In some cases, these viruses can also replicate in the vector’s body [32,34]. The quantitative analyses of viral RNAs in developmental stages of fungus gnat showed that the amount of viral RNA1 encoding 1a ORF in the pupa stage was in a few cases higher than in the larvae that acquired viral particles. The obtained data suggested a far-reaching hypothesis that PSV may circulate or even replicate in fungus gnats tissues in contrast to the above-mentioned transmission in the aphid. On the other hand, the differences in virus RNA accumulation are an individual matter of specimens, and the larvae (from which the analysed pupae developed) previously acquired much more virus compared to the others. However, only further experiments will shed light on the replication of this virus in different developmental stages of *B. impatiens*.

On the basis of the available literature, *Bradysia* species are known to be vectoring soil-borne pathogens [21]. In 1990 Gardiner et al. showed that the larval stage of *B. impatiens* might act as an important vector for *Pythium* spp. [21]. However, there are no literature data on the role of *Bradysia* spp. in virus spreading. The transmission assay performed in this study has shown that *B. impatiens* larvae exposed to PSV are able to transmit the virus to a healthy plant. In 2010, Braun et al. have reported transstadial transmission of *Pythium* and proved the lack of *B. impatiens* adults vectoring ability [15]. The vectoring capacity of adult stages has been poorly studied. Nevertheless, a few reports have indicated the role of adults of *B. impatiens* in pathogen transmission [12]. Kapongo and co-workers in 2020 showed that not only larva, but also adults of *B. impatiens* might be a vector of *Fusarium oxysporum* and *Pythium aphanidermatum* [35]. In this study, we have not confirmed the ability of an adult insect to transmit the virus to healthy plants. On the basis of the obtained data, the virus acquired in the larval stage persists transstadially, which is also in agreement with the previous report published by Braun and co-workers [15]. Hence, the most likely possibility is that a virus is transmitted by the larva in the second generation (F2). As mentioned above, PSV was detected in the eggs and larvae of the F2 generation, but only in one experiment. In general, we suppose that virus might be carried on a very low level in F2 eggs or might be present only in some of them, or it may not always be transferred to the next generation. Hence, this issue needs further experiments that will include a larger number of samples, to draw reliable conclusions.

In the molecular experiments complemented with biological test on PSV-exposed larvae and healthy plants of *N. benthamiana*, we have shown that *B. impatiens* larvae have the vectoring capacity. However, after the inoculation access period, the level of the viral RNA accumulation in plant tissues, in almost all cases, was rather low. Visual observation of plants inoculated by viruliferous *B. impatiens* revealed no characteristic symptoms of PSV infection. The typical symptoms of PSV infection on *N. benthamiana* are stunting, leaves malformation, and chlorosis. However, in some cases, the symptoms may be attenuated, depending on various conditions, among other temperature [22,36]. Herein, the symptomless infection might result from a small number of virus particles or low transmission efficiency caused by a small number of insect specimens used in these experiments. However, using the Western blotting approach, we have confirmed the presence of viral coat protein, which proves that the genomic strands of PSV, detected in the plants exposed to viruliferous larvae of *B. impatiens*, are infectious.

Thus, on the basis of the above-described findings, *B. impatiens* role as a plant virus vector, or a reservoir of this pathogen, should be taken into account. The obtained data help to better understand the biology of *B. impatiens* and its role in plant pathogen spreading.

## Figures and Tables

**Table 1:** The sequences of diagnostic primers hybridizing to peanut stunt virus RNAs.

Genomic Strand	Primer	Sequence 5′> 3′		Amplicon Length, Annealing Temperature Ta °C
RNA1	P1a	CACAAATCCGGCTGAGAAATG	this study	426 bp, 57.3
	P1b	CAAGATACCAGCGTAGATCAC		
	q1F	CTTCTGCCCTCGTTGATAAAG	[22]	131bp, 57
	q1R	CATACCGATTTCGAATCACTTC		
RNA2	v2b1	TGAGAATTCAAAAAAAAAACAATGTCGAG TGTCGAGCAG	this study	320 bp, 59
	v2b2	CGCCTGCAGTTATCAGGAATAACTACCCTC		
	q2aF	CTTCTAGGTATCCCCGTAAG	[22]	130 bp, 56
	q2aR	CAAGCACATTGATACCCTATC		
RNA3	P3MPCP1	GAGGTATGGTTATCTTGGACATC	[19]	958 bp, 60
	P3MPCP2	GAAGTTGAACACAGGAAACCTTC		
	qCP F	ACACATACACTTCGTTGGATG	[22]	130 bp, 56
	qCP R	CCTCWTCTTCGGAAATTCAG		

## Data Availability

All data are provided in the manuscript and in the GenBank database.

## Supplementary Materials

The following are available online at https://www.mdpi.com/article/10.3390/cells10061546/s1, Figure S1: RT-PCR detection of PSV 1a gene of chosen developmental stages of *Bradysia impatiens*.
