# Supplementary figures and images for: Analysis of the Role of Bradysia impatiens (Diptera: Sciaridae) as a Vector Transmitting Peanut Stunt Virus on the Model Plant Nicotiana benthamiana
Source: Cells. 2021 Jun 18;10(6):1546. doi: 10.3390/cells10061546 (PMC8233879; doi:10.3390/cells10061546)

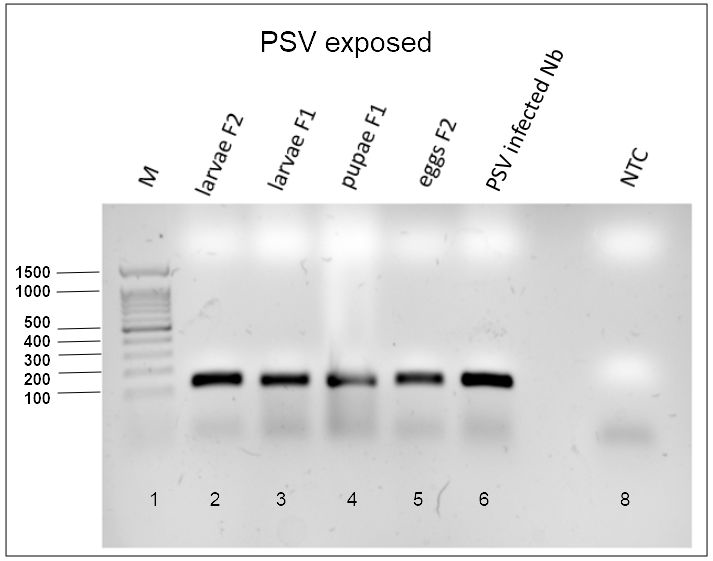

Supplement: Supplementary file 1 [file cells-10-01546-s001.zip › cells-1171635-s1.tif]
